# Lessons learned from independent external validation of an AI tool to detect breast cancer using a representative UK data set

**DOI:** 10.1259/bjr.20211104

**Published:** 2023-02-06

**Authors:** Dominic Cushnan, Kenneth C Young, Dominic Ward, Mark D Halling-Brown, Stephen Duffy, Rosalind Given-Wilson, Matthew G Wallis, Louise Wilkinson, Iain Lyburn, Richard Sidebottom, Rita McAvinchey, Emma B Lewis, Alistair Mackenzie, Lucy M Warren

**Affiliations:** 1 AI Lab, NHSX, Skipton House, London, United Kingdom; 2 Royal Surrey NHS Foundation Trust, Guildford, United Kingdom; 3 University of Surrey, Guildford, United Kingdom; 4 Queen Mary University London, London, United Kingdom; 5 St George’s Healthcare NHS Trust, Tooting, London, United Kingdom; 6 Cambridge Breast Unit and NIHR Cambridge Biomedical Research Centre, Cambridge University Hospitals NHS Trust, Cambridge, United Kingdom; 7 Oxford University Hospitals NHS Foundation Trust, Oxford, United Kingdom; 8 Gloucestershire Hospitals NHS Foundation Trust, Cheltenham, United Kingdom; 9 Cobalt Medical Charity, Cheltenham, United Kingdom; 10 Cranfield University, Shrivenham Campus, Cranfield, United Kingdom; 11 The Royal Marsden NHS Foundation Trust, Surrey, United Kingdom; 12 Jarvis Breast Screening Centre, Guildford, United Kingdom

## Abstract

**Objective:**

To pilot a process for the independent external validation of an artificial intelligence (AI) tool to detect breast cancer using data from the NHS breast screening programme (NHSBSP).

**Methods:**

A representative data set of mammography images from 26,000 women attending 2 NHS screening centres, and an enriched data set of 2054 positive cases were used from the OPTIMAM image database. The use case of the AI tool was the replacement of the first or second human reader. The performance of the AI tool was compared to that of human readers in the NHSBSP.

**Results:**

Recommendations for future external validations of AI tools to detect breast cancer are provided. The tool recalled different breast cancers to the human readers. This study showed the importance of testing AI tools on all types of cases (including non-standard) and the clarity of any warning messages. The acceptable difference in sensitivity and specificity between the AI tool and human readers should be determined. Any information vital for the clinical application should be a required output for the AI tool. It is recommended that the interaction of radiologists with the AI tool, and the effect of the AI tool on arbitration be investigated prior to clinical use.

**Conclusion:**

This pilot demonstrated several lessons for future independent external validation of AI tools for breast cancer detection.

**Advances in knowledge:**

Knowledge has been gained towards best practice procedures for performing independent external validations of AI tools for the detection of breast cancer using data from the NHS Breast Screening Programme.

## Introduction

**Figure 1.**
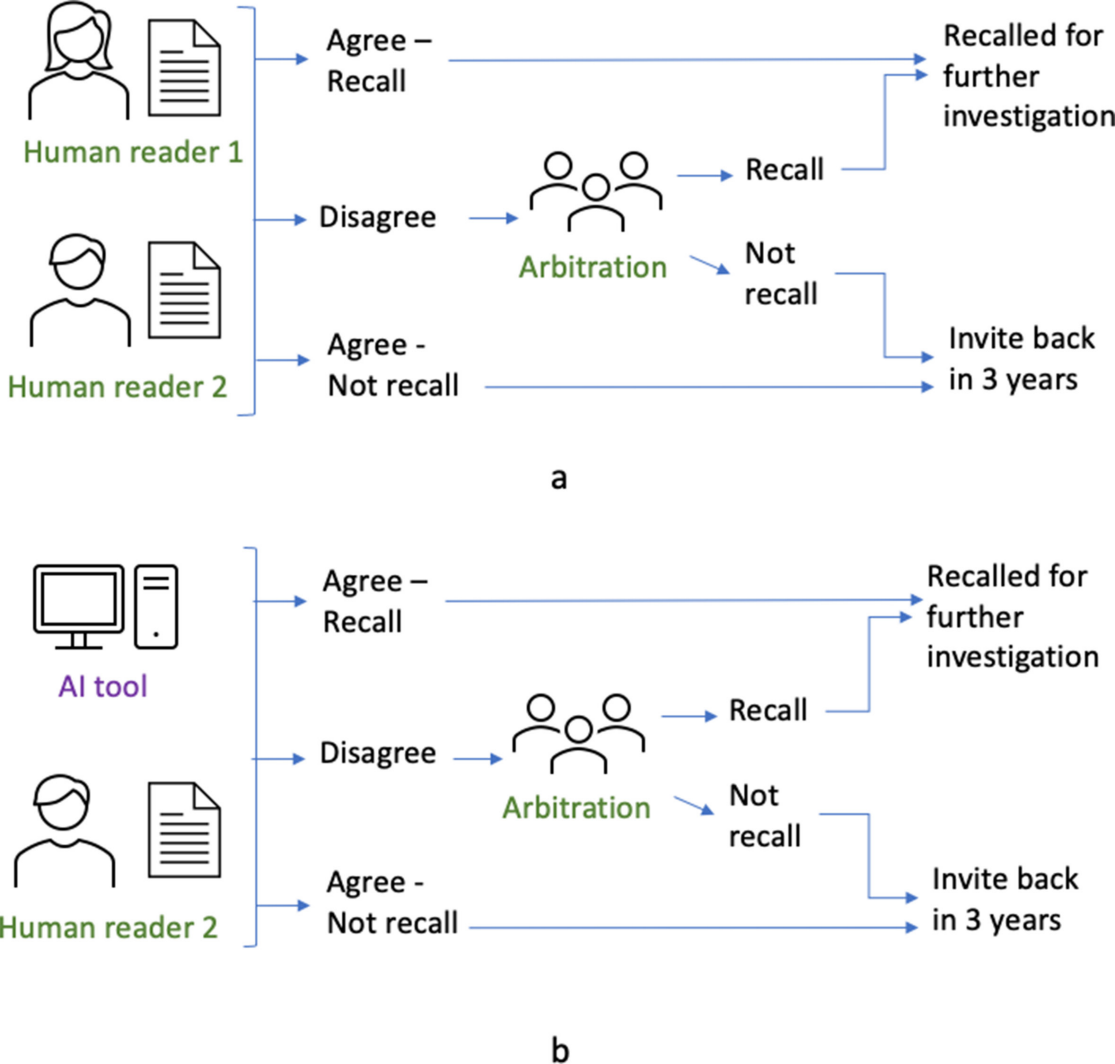
(**a**) Normal screening workflow. (**b**) Workflow when AI tool replaces one of the human readers (independent AI reader). AI, artificial intelligence.

Mammography is the standard imaging modality used in population breast screening worldwide, including in the NHS Breast Screening Programme (NHSBSP) in the UK. In the NHSBSP, females from the ages of 50 up to their 71st birthday are invited to attend mammographic screening every 3 years. Mammograms for each female are interpreted by two readers (termed double reading). There is some variation in practice across the NHSBSP so that depending upon the screening centre, the second reader interpretation is either blinded or unblinded.^1^ Arbitration will occur if there is disagreement, or in some screening centres for all recalled females as well as disagreements (Figure 1a). Around one in four breast cancers in the screened population are not detected at screening, but symptomatically between screening rounds (termed interval cancers).^2^

AI is an attractive opportunity for breast screening, with the potential to help manage the high workload, to improve the detection of breast cancers, and to reduce the number of interval cancers. AI tools could be integrated into the NHSBSP at several stages in the pathway. Examples include replacement of either the first or second human reader, and triage of females to be read by human reader, or as a decision-support tool. In this evaluation, the AI acted as the replacement of the first or second human reader (Figure 1b) and its performance was compared to human readers. The exercise did not study the interaction of human readers with the AI tool, *e.g*. at arbitration.

Developers of AI tools use training and validation data sets to tune the hyperparameters of their tools. Once trained, they may internally validate their AI tool on separate test data. Without independent external validation on a representative data set, tools may not perform as expected in real-world clinical settings with heterogeneous sociodemographic settings. A clear definition of the processes and minimum requirements to validate AI tools for Health and Social Care use would help build trust, which is paramount for the wide adoption of safe and effective new tools, and provide an accelerated pathway to deployment.

Previous internal and external validations have reported that AI tools can approach the performance of radiologists using retrospective data for breast screening.^3–5^ In order to provide a fair comparison, it is essential that data on screen detected and interval cancers are available, but only a few publicly accessible databases exist to enable such a comparison.^6,7^ A recent NICE Medtech Innovation briefing^8^ found that a key problem was the lack of clinical validation studies using UK data sets representative of the target population for screening. Additionally, Freeman et al^9^ highlighted the need for more evidence prior to the implementation of AI into clinical practice.

Public Health England (PHE) and NHSX collaborated with the OPTIMAM Breast AI Team, to address this limitation by piloting an independent external validation process, to understand its challenges and complexities, and to work towards the definition of best practice and standard procedures. In the work presented here, considerations and recommendations for an external validation of an AI tool are discussed. Note that the details and performance of the particular AI tool used in this study are not disclosed, instead the focus is on lessons learned in creating tools for best practices and standardisation for any medical imaging AI validation.

## Methods and materials

### Source of data

This retrospective study used images from the OPTIMAM mammography image database (OMI-DB).^7^ All images and data in OMI-DB are de-identified. A proportion of the images have been shared with research and commercial groups for training and validation of AI algorithms and a record is kept of the sharing history.

OMI-DB contains data for females screened at the Jarvis Breast Screening Centre (Guildford) and St George’s Hospital (South West London) from 2011. Images and data for all females screened in 2014 have been collected. For females screened in other years since 2011, all females who had cancer diagnosed (screen detected or interval cancer) were collected and a proportion (25%) of females without cancer were collected. Any new images or clinical data for these females are continuously added. For this study, follow-up data were included up to June 2020.

OMI-DB also contains clinical and pathological information from the National Breast Screening System (NBSS). Ethnicity is populated for 94% of females in OMI-DB and the distribution is shown in Supplementary Appendix 1.

Supplementary Material 1.

### Data sets

Two data sets were created, a representative data set and an enriched positive data set. The representative data set aims to have the same characteristics (such as cancer rates, ethnicity, age), as all females attending screening at the Jarvis and St George’s breast screening centres in 2013–2015. The enriched positive data set is a data set of only positive cases. It does not aim to be representative, but provide a large enough number of positive cases, in order to have sufficient statistical power for each type of positive case—screen-detected cancer, prior to interval cancer and prior to screen-detected cancer at the next screen. Images from some females in OMI-DB had previously been shared with the AI vendor. These females were not included in either data set.

### Representative data set

The screening mammograms for 26,000 females were selected to be representative of all females attending screening at the Jarvis and St George’s breast screening centres in 2013–2015. Full details of the selection process are described in Supplementary Appendix 2. The representative data set (from two centres) have descriptive statistics similar to those of a single year of the OMI-DB repository as given in Table 1. In this representative data set of 26,000 females, 526 (2.0%) had breast cancer diagnosed within 39 months of the screening episode. This time period allows for almost all cancers potentially detectable at the index screening event to be included. These 526 females include 253 females with a screen-detected cancer detected in the index screening episode, and 84 and 189 females respectively with an interval cancer or screen-detected cancer diagnosed within 39 months of the normal index screening episode.

**Table 1.** Characteristics of the representative data set and single complete year in OMI-DB (2014)

Characteristic	Category	Representative data set (2012–2015)		OMI-DB (2014)	
		n	(%)	n	(%)
Age	50 to <55 55 to <60 60 to <65 65 to <71	7573 6354 5972 6101	29 24 23 24	24,522 20,752 18,770 20,461	29 25 22 24
Ethnicity	White Mixed Asian Black Other Not recorded	21,622 755 1197 622 1204 600	83 3 5 2 5 2	68,201 2689 3832 1941 4087 3755	81 3 5 2 5 4
Breast thickness (Average per study)	<20 mm 20–40 mm 40–60 mm 60–80 mm 80–100 mm 100 + mm	43 2578 12,456 10,313 607 3	0 10 48 40 2 0	156 8417 39,988 33,777 2142 25	0 10 47 40 3 0
Manufacturer	Siemens GE Hologic	835 957 24,208	3 4 93	2076 3199 79,230	2 4 94

OMI-DB, OPTIMAM mammography image database.

### Enriched positive dataset

To select the enriched data set (Table 2), all screening mammograms, for females aged 50–70 years, in which cancers were detected, those prior to interval cancer occurrence and those prior to a screen-detected cancer from 2011 to 2020 at the two screening centres in OMI-DB were identified. Females in the representative data set and females whose images had previously been shared with the vendor were excluded. The data set was used to evaluate the effect of cancer grade on the sensitivity of the AI tool. It included 1589 females with 2052 screening episodes. From the 1589 women, 1126 had one episode and 463 had two episodes—one “prior to screen-detected cancer” episode and one “screen-detected cancer” episode. The date difference between these ranged from 12 to 39 months. However, the vast majority (92%) were between 30 and 39 months. The episodes included:

- 1049 episodes with a screen-detected cancer
- 241 episodes prior to occurrence of an interval cancer
- 762 episodes prior to a screen-detected cancer.

**Table 2.** Characteristics of the enriched positive data set

Characteristic	Category	Enriched positive data set	
		n	(%)
Age	50 to <55 55 to <60 60 to <65 65 to <71	595 482 470 505	29 23 23 25
Ethnicity	White Mixed Asian Black Other Not recorded	1562 49 87 38 99 217	76 2 4 2 5 11
Breast thickness (Average per study)	<20 mm 20–40 mm 40–60 mm 60–80 mm 80–100 mm 100 + mm	2 212 874 886 77 1	0 10 43 43 4 0
Manufacturer	Siemens GE Hologic	77 56 1919	4 3 93

### AI tool

The intended use of the AI tool applied in this validation exercise is to aid readers in the interpretation of breast imaging examinations for the early detection and diagnosis of breast cancer as one of the readers in blinded or unblinded workflows. The exclusion criteria for the AI tool were male patients, unprocessed images, magnified images, females with previous surgery, images after breast cancer diagnosis, females with implants and studies with more or fewer than four images.

Two operating points were requested from the AI vendor prior to the study:

- Study specified operating point: operating point at a target specificity set equal to the average second reader specificity in OMI-DB.
- Vendor specified operating point: operating point recommended by vendor if installing clinically.

The AI tool generated a binary decision for each female (cancer/no cancer), for each operating point.

### Deployment and security

The AI tool was deployed on cloud infrastructure. Details on the deployment and security settings are provided in Supplementary Appendix 3.

### Statistical analysis

#### Definition of positive and negative case

A positive case was defined as a female diagnosed with breast cancer (interval cancer or screen-detected cancer) within 39 months of the screening mammogram based on pathological information. A negative case was defined as a female whose mammograms were read as normal by human readers at the time of screening and had a normal follow-up mammogram at least 20 months after the study mammograms. Further details are given in Supplementary Appendix 2.

#### Statistical tests—representative data set

The study assessed non-inferiority of the AI system to human readers. The sensitivity and specificity of both the AI and the human readers were calculated using McNemar analysis.^10^ A non-inferiority range of 5 percentage points was chosen (this is an absolute difference—*e.g.* going from a specificity of 92% to 87% would be a decrease of 5 percentage points). If the lower point of the 95% confidence interval in the difference is less than the non-inferiority range, the AI product is non-inferior to the human reader. A receiver operating characteristic curve of the AI performance was generated. In addition, the positive-predictive values and negative-predictive values were calculated at the operating points. The percentage of females where a disagreement occurred between the AI and human readers was calculated. Finally, the rate at which the first reader, second reader, consensus and AI would recommend recall were calculated.

#### Statistical tests—enriched data set

Using the enriched data set, the sensitivity of the AI tool was calculated by type of positive case (screen-detected cancer, prior to screen-detected cancer, and prior to interval cancer). For screen-detected cancers, the sensitivity of the AI tool was compared to human readers for each grade of cancer and invasive status were compared to the human readers.

### Sample size

Power calculations prior to the study indicated that 470 cancers as used in this study would have 90% power with a non-inferiority range of 10 percentage points, and 80% power with a non-inferiority range of 5 percentage points.

## Results—lessons learned

### Rates of disagreement between readers and AI tool

The rate at which females were recalled by the AI tool was the same as the second human reader when operating at the target specificity. There was a higher rate of disagreement between the AI tool and the first human reader, than the rate of disagreement between the second and first human readers. Variations in practice at different centres (*e.g.* whether second reader is blinded to first reader’s opinion, and which cases go to arbitration) will affect the rate of disagreement between human readers. The disagreement was higher, for the same recall rate, because the AI tool did not not recall some screen-detected cancers recalled by humans, but recalled some prior to interval and screen-detected cancers not recalled by either human reader. While it may be beneficial that the AI tool is recalling different cancers to the human readers, it will increase the number of cases sent to arbitration. Arbitration is a review of the more complex cases, which is usually much more time consuming than first or second reading. The next step after a technical validation, as described here, would be a clinical validation of the impact of AI on the decisions made by radiologists during arbitration. This could be achieved using virtual clinical trials or a clinical study.

### Outputs of the AI tool

When designing this validation, the AI vendor was asked that their AI tool provide both a continuous score or probability for each female and the location of identified lesions. The AI vendor did not wish to provide this information for this validation exercise.

This validation highlighted that locations for AI detected abnormalities are necessary to understand whether the AI tool is recalling the same cancer as the human reader or an early interval or screen-detected cancer and these should be required in future validations. The need for location information is dependent upon the clinical application, and for example will be important at arbitration and at assessment but may not be essential if using an AI tool for triage. The clinical need for location information should dictate whether the location information provided by AI tools is evaluated in future validations.

### Selection of operating point for validations and target specificity

The ROC curve demonstrated the trade-off between sensitivity and specificity when changing operating point, and therefore the large clinical impact of the choice of operating point. It is essential to validate the AI tool in a setting that is as close to the clinical use as possible. Two operating points were used for this study. One selected by the vendor and the other selected to be at a target specificity equal to the average second human reader specificity in the centres included in OMI-DB. The latter operating point was defined based on specificity as it was felt by the radiologist advisors that an increase in recall rate would not be manageable to the screening programme.

The operating point at a target specificity was used because this allowed a direct comparison of sensitivity of the AI tool at the same specificity as the human readers. When the AI tool was used at the operating point selected by the vendor, it had significantly higher sensitivity and lower specificity. It is likely that the specificity of first and second reader and arbitration varies between units and with reading protocols. It is not known what the desirable operating point would be, once arbitration is taken into account. In addition, it will depend on the importance attached to very high sensitivity *vs* acceptable specificity, which may vary in different healthcare contexts (*e.g.* screening or symptomatic services).

### Images rejected by AI

A validation data set needs to include the full range of images and females seen in screening, to measure how these are handled. Future validations should provide sufficient information on images rejected by the AI tool so that services can decide whether the tool works for them, in terms of the impact on workload and clinical benefit. The clarity and accuracy of error messages should be evaluated as in this study.

### Technical recalls

When evaluating AI tools, it should be determined whether the AI tool can either correctly identify whether images are technically adequate for diagnostic reporting or reject the female’s screening episode. Either would probably be preferable to processing technically inadequate images. An enriched data set of technically suboptimal images could be used to test whether the AI tool could operate using images that would be rejected by a human.

### Data set selection

The main data set was selected to be representative of the females attending the two screening centres in the study. In addition, an enriched data set of positive cases allowed the performance of the AI tool to be evaluated by grade of cancer and invasive status.

To investigate the performance of the AI tool on specific subgroups such as ethnicity, age, radiological appearance, cancer pathology and X-ray equipment manufacturer, large enough numbers of females would be required in each subgroup to achieve statistical significance. Therefore, additional data sets could be collected, where these groups are not representative but enriched to allow subgroup analysis. In addition, the pathological data should be well populated in order to validate the AI tools on different cancer types.

### Definition of a positive case

The definition of a positive case in the validation study is relevant to the 3 year screening round used in the UK breast screening programme. Positive cases include screen-detected cancers, prior images to interval cancers and prior images to screen-detected cancers. It provided the AI tool the possibility of detecting cancers up to 39 months earlier than found later by the screening programme or appearing symptomatically within this time period. This is regarded as a fair test as the human readers had the same opportunity and is similar to the approach adopted in McKinney et al.^2^ This is only a fair test if the rate of screen-detected cancers, interval cancers and prior to screen-detected cancers are as seen clinically, as was ensured in this study. The AI tool will be favoured if the validation data set includes a disproportionate number of interval or next round screen detected cancers compared to real life screening. Conversely, the human reader will be favoured if there is an excess of screen detected cancers. Complete follow-up data are therefore important when validating AI tools.

### Interval for non-inferiority

In non-inferiority testing, a range for the possible true difference between the treatments is defined.^9^ If every point within this range corresponds to a difference of no clinical importance, then the treatments may be considered to be equivalent. Traditionally, a range of 10 percentage points is used in such studies.^11^ However, for breast screening it was decided that 5 percentage points, whilst potentially still too large, was more appropriate, since 10 percentage points would be a larger clinically impactful difference in performance. Other studies evaluating AI performance have also used 5 percentage points for the non-inferiority range.^3^ Prior to future validations, discussions will be needed on what is an acceptable difference in sensitivity and specificity. This can then inform sample size estimations for such studies and be used for the non-inferiority tests.

### Clinical input

Evaluating an AI tool involves several stages for which there are multiple options potentially affecting the outcome of the validation. An iterative approach was used to develop the methodology, with each step discussed with the radiologists on the Breast AI Evaluation Team. A clinical team’s involvement is essential at all stages of the validation of an AI tool.

### Discussion

This study demonstrated how retrospective data from the NHSBSP can be used to quantify the ability of AI tools to recall cancers in the UK breast screening population, and found lessons for future validations, listed below. The availability of individual readers’ and arbitration opinions and long-term follow up with collection of interval cancer cases and subsequent screening data allowed comparison with real-world data. A limitation of this study was the lack of adequate numbers of images across the range of X-ray manufacturers in use across the NHSBSP. This can be solved by recruiting more screening centres for data collection.

The gold-standard method of comparing the performance of an AI tool to human readers would be a prospective randomised controlled trial. However, these are time-consuming and costly. This is complicated by the fact that new versions of existing AI tools are likely to be released in the future. Therefore, although a trial may be performed to initially evaluate the technology, performing a trial for every vendor, version and prototype is impractical. It is therefore essential for NHS programmes to have the ability to conduct a rapid, robust evaluation of new tools that come to the market, as well as updates to existing ones. This work compared the performance of an AI tool as an independent reader to human readers using a retrospective data set. Four major unanswered questions include:

1. What is the impact of operating point selection on cancer detection and recall rate after arbitration?
2. What is the impact of AI tool decisions on arbitration meetings?
3. What is the impact of AI suggested recalls on the assessment process?
4. What is the impact of using AI tools on mortality from breast cancer?

To answer these questions in a prospective trial would take many years, since trials would need to run for at least 3 years to collect subsequent interval cancers and cancers detected in the next round of screening. We recommend conducting virtual clinical trials using retrospective data sets to answer these questions. Such virtual clinical trials are needed because running the AI tool on retrospective data alone cannot evaluate how human readers will react to information from these new AI tools. For instance, whether readers at arbitration would correctly recall an interval cancer identified by AI but not the human reader.

### Recommendations for future retrospective independent external validation of AI tools in breast screening

#### Recommendations related to general validation process

- External retrospective validation studies should be the first stage of independent validation, performed to quantify the technical performance of an AI tool prior to prospective clinical evaluation.
- After independent technical external validation, virtual clinical trials investigating the interaction of human readers with the AI tool should be performed prior to prospective clinical evaluation to measure the impact of the AI after arbitration
- The clinical use-case of the AI tool is vital for the design of the validation. For example, if the clinical requirement is that the AI tool provides location information, then the output of such location information should be a necessary requirement in order for the external validation of the AI tool to be performed.
- A clinical team should be involved in all stages of the validation.
- Minimum requirements should be set for external validation of AI tools for NHS use.

#### Recommendations related to data sets

- Data sets used for retrospective validation studies should be representative of the screening population and have sufficient follow-up data to include a representative number of interval cancers and next round screen-detected cancers.
- Data sets should not be curated to meet the exclusion criteria of the AI product so that the performance of the AI tool on all types of cases seen clinically is evaluated.
- Enriched data sets should also be used in retrospective studies to facilitate analysis of the performance of AI tools on minority subgroups in a representative data set, such as X-ray system manufacturer, ethnicity groups, types of breast cancer or technical recalls.
- The data set should be large enough for sufficient statistical power at the agreed non-inferiority range.

#### Recommendations related to statistical analysis

- A non-inferiority range that is clinically relevant for breast screening needs to be decided.
- The performance of the AI tool with type of positive case (screen-detected cancer, prior to interval cancer and prior to screen-detected cancer) should be determined. Reducing symptomatic presentation between screens (*i.e.* interval cancers) may be a good proxy for detecting important clinical cancers and achieving a mortality benefit.
- The performance of the AI tool should be evaluated at the operating point the AI vendor would recommend clinically. In addition, clarity is needed as to whether the operating point should be defined by the service or the AI tool provider. There would be additional value in also testing tools at a standard operating point with an agreed target specificity similar to human readers for ease of comparison to humans and other AI tools.
- The level of rejection of images by the AI tools which would be clinically acceptable and can be handled in a different workflow should be decided. The clarity of warning messages for such rejected images should be assessed as part of validation.

## Conclusion

This study demonstrated how a retrospective independent external validation of AI tools for breast cancer can be conducted using a representative data set from OMI-DB and provides recommendations for future external validation.
