# Supplementary material for: Lessons learned from independent external validation of an AI tool to detect breast cancer using a representative UK data set
Source: Br J Radiol. 2023 Feb 6;96(1143):20211104. doi: 10.1259/bjr.20211104 (PMC9975375; doi:10.1259/bjr.20211104)
Supplement: Supplementary Material 1. [file bjr.20211104.suppl-01.pdf]

Appendices

Appendix 1 - Additional information on the images and data in OMI-DB

Additional information on the images and data in OMI-DB can be found in the table below. Statistics are provided for all women screened in 2014 at the two screening centres.

Appendix Table 1.1 Table of characteristics of OMI-DB

	**OMI-DB statistics**	
**Breast Screening Service**	Guildford	South West London
**Time period**	2014	2014
**Total number of women screened (aged 50-70)**	42742	41763
**No of prevalent women screened**	5161	8759
**No of incident incident women screened**	37581	33004
**No of prevalent women screened 50-52[1]**	3716 (87.2)	6812 (163.1)
**No of incident women screened 53-<71[1]**	34089 (797.3)	31986 (765.9)
**Mean age of women screened (years)**	59.4	58.8

**Distribution of ethnicity [2,3]**	NULL 482 (1.13)<br>A 37423 (87.6)<br>B 444 (1.03)<br>C 1502 (3.5)<br>D 55 (0.12)<br>E 23 (0.05)<br>F 88 (0.21)<br>G 63 (0.15)<br>H 526 (1.23)<br>J 100 (0.23)<br>K 29 (0.07)<br>L 251 (0.59)<br>M 84 (0.20)<br>N 134 (0.31)<br>P 23 (0.05)<br>R 305 (0.71)<br>S 794 (1.85)<br>Z 416 (0.97)	NULL 3273 (7.84)<br>A 25209 (60.4)<br>B 908 (2.17)<br>C 2484 (5.95)<br>D 176 (0.42)<br>E 94 (0.23)<br>F 221 (0.53)<br>G 157 (0.38)<br>H 1517 (3.63)<br>J 497 (1.19)<br>K 125 (0.30)<br>L 1414 (3.39)<br>M 1332 (3.19)<br>N 863 (2.07)<br>P 88 (0.21)<br>R 528 (1.26)<br>S 425 (1.02)<br>Z 2452 (5.87)
**Assessment rate (%)**	5.05	5.28
**No of women recalled to assessment from the prevalent round[2]**	563 (10.9)	890 (13.1)
**No of women recalled to assessment from the incident round[2]**	1597 (4.25)	1314 (4.11)
**Screen-detected cancer detection rate (per 1000 women screened)**	9.29	9.72
**Percentage of cancers which are invasive (%)**	76.8	73.7
**No of invasive cancers detected from the prevalent round**	33	68
**No of invasive cancers detected from the incident round**	272	232

**No of non-invasive cancers detected from the prevalent round**	17	28
**No of non-invasive cancers detected from the incident round**	75	79
**Grade distribution of invasive cancers detected from the prevalent round[4,5]**	G1 8<br>G2 19<br>G3 5<br><br>NULL 1	G1 23<br>G2 26<br>G3 17<br>NA 1<br>NULL 1
**Grade distribution of invasive cancers detected from the incident round[4,5]**	G1 75<br>G2 147<br>G3 46<br>NULL 4	G1 46<br>G2 140<br>G3 41<br>NA 5
**Grade distribution of non-invasive cancers detected from the prevalent round[4,5]**	NDH 6<br>NDI 4<br>NDL 4<br>NULL 3	NDH 14<br>NDI 9<br><br>NULL 5
**Grade distribution of non-invasive cancers detected from the incident round[4,5]**	NDH 42<br>NDI 18<br>NDL 7<br>NULL 8	NDH 43<br>NDI 27<br>NDL 5<br>NULL 4

[1]Bracketed values are rates per 1000 women screened.

[2]Bracketed values are percentages.

[3]Codes for Ethnicity are taken from NBSS, key is available here:

https://www.datadictionary.nhs.uk/data_dictionary/attributes/e/end/ethnic_category_code_2001_de.asp?shownav=1
[4]NA = Not assessable
[5]G1 = Grade 1, G2 = Grade 2, G3 = Grade3, NDL = Low grade, NDI = Intermediate grade, NDH = high grade

Appendix 2 – Selection process

The selection process is summarised below:

- Randomly select 26000 screened women from 2013-2015 from two screening sites in OMI-DB
- Eligibility criteria:
  - Aged 50 to <71
  - Had a general breast screening episode (excluding all women who attended high risk screening)
  - The screening episode includes a screening event in NBSS and mammography images collected in OMI-DB
- Images and data from OMI-DB are shared widely with research groups and commercial companies developing AI solutions. It is important that no images that have been used to train the AI product are used to validate its performance. Therefore, it was important to replace any of the above women whose images had been shared with the AI vendor with those not shared with the AI vendor. When replacing women they were matched using the following criteria:
  - Woman same age (closest possible)
  - Same breast thickness (closest possible)
  - Whether the screening images were from the first screening round or not
  - Same screening outcome
  - For cancers same:
    - Invasive status

In the long term such replacements will not be necessary since the OPTIMAM team is in the process of collecting a large dataset which will not be shared with any AI developer.

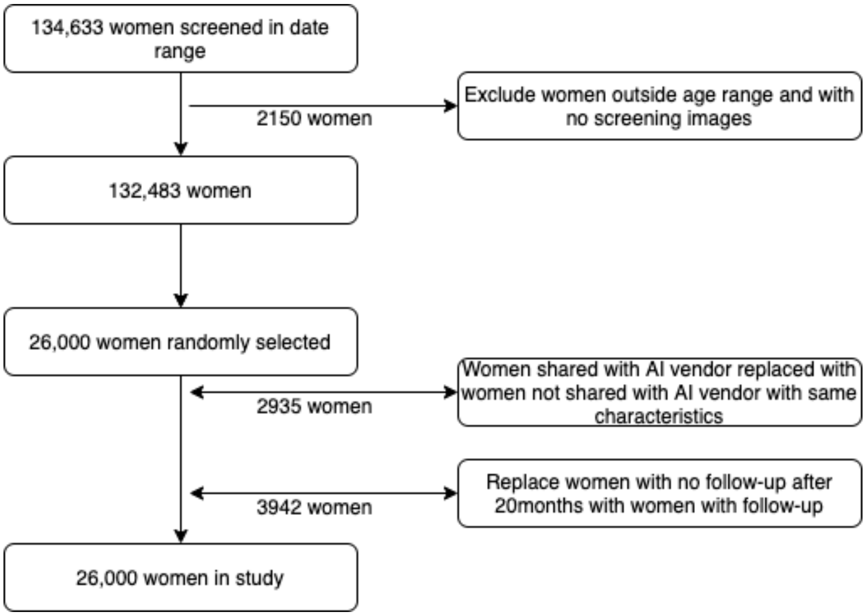

Appendix Figure 1. Flowchart showing selection of dataset

The number of interval cancers in NBSS at each of the two screening centres was compared to those recorded in the screening histories information management system (SHIM). The number of interval cancers which should be present in the sample of 26000 women was calculated from the rate of interval cancers recorded in SHIM for these two centres. This target number of prior to interval cancers in the sample of 26000 women was achieved by randomly sampling from all prior to interval cancers in the year range 2013-2015.

If a woman attended screening more than once in the study period, the screening episode included was randomly selected. 1.6% of women attended more than once in the date range considered in this study. Some women with no cancer found at screening may not return for the next screening round. These women were replaced with women with the same characteristics (as was done with the cases which had been shared with the vendor), such that all women with no cancer had a normal follow-up mammogram at least 20 months after the study mammograms. The prevalence of cancer, distributions of ethnicity and age, proportion of episodes which were first screen and rate of assessment and biopsy were not changed by this replacement.

Appendix 3 – AI Validation Cloud Deployment Details

This appendix describes the details of the cloud deployment and security settings utilised for the AI validation project

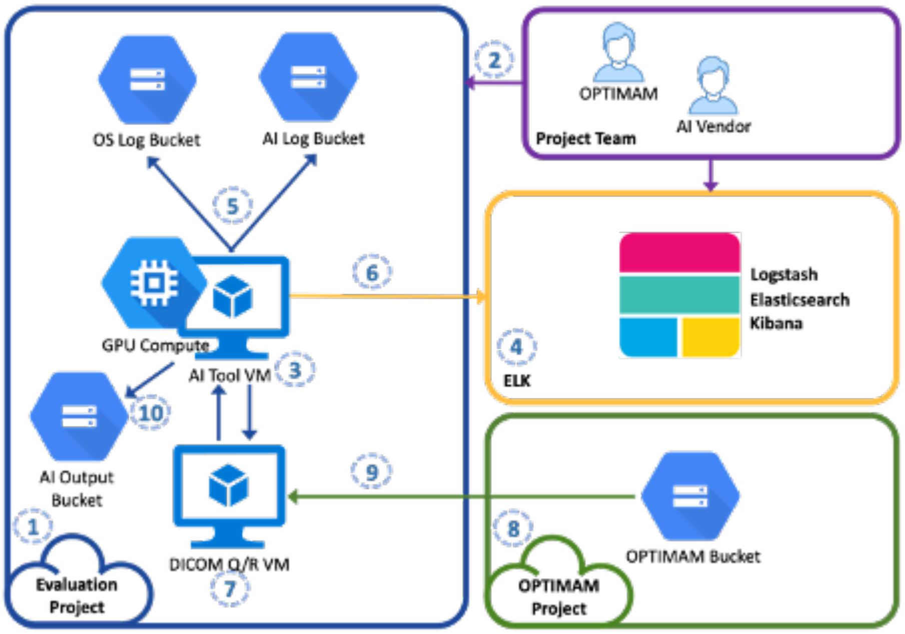

Appendix Figure 2. Overview of AI validation cloud deployment

1. **Dedicated Cloud Project:** A dedicated cloud project was created for the AI validation project on the Google Cloud Platform (GCP). Project security included:
    1. Limiting the use of Cloud IAM primitive roles
    2. Securely connecting to Virtual Machine (VM) instances (https://cloud.google.com/solutions/connecting-securely)
    3. Ensure firewall rules were not overly permissive, with principle of least privilege and default deny, and a minimal set of allowed protocols and ports
    4. Enable VPC Flow Logs
    5. Enable and configure Stackdriver logging and monitoring
2. **User Access**: the number of users were kept to a minimum and access restricted to named project team members only. IAM groups and roles were used to provide simple configuration of access control policies enabling granular access to resources. These were setup to permit access for users and groups to specific resources only where necessary, and where feasible included restricting access based on date/time and IP address. IAM access controls were always configured to be as restrictive as possible.
3. **Dedicated VM for AI Tool**: Separate VM for hosting AI tool including OS system logging via the use of AuditD. AuditD was configured to be compliant with Controlled Access Protection Profile, and commands logged included, but not be limited to: `crond auth sshd sudo xtables-multi systemd passwd`. In order to interpret the logs, the following tools could be used: `ausearch` (which enables searching for particular events in the logs using search criteria like user, event type, process etc), and `aureport`, which can generate summary information from the logs. These tools were deployed on the VM, and scheduled to be run at a suitable interval. Output was synced to the monitoring bucket (‘OS Log Bucket’); in addition a regular sync of the log-file itself to the

bucket was scheduled. In this instance the VM OS was CentoOS 7 and was provisioned with 32 GB RAM and 8 vCPU.

4. **ELK Stack Log Viewer**: Externally accessible ELK stack which members of the AI Vendor project team were able to access the OS logs.
5. **Logging Buckets:** Separate buckets for logs to be written to, visible to all project team members
    1. OS logs in the form of Auditd logs - Accessible to all project team members.
    2. AI Tool logs (bespoke) - Accessible to all project members, however NOT accessible to AI vendor team members during the running of the validation process.
6. **OS Logs routing to ELK Stack:** Logs shipped to logz.io**.** Configuration file for log shipping tools accessible to the AI Vendor project team. The logs that are shipped are: AuditD and VPC logs.
7. **VM for DICOM Q/R:** A Centos GPC VM was created, which facilitated the sending of images to the AI tool and the receiving of the DICOM formatted AI tool outputs. This VM was a minimal configuration Centos 7 with 2vCPU and 4GB RAM.
8. **OPTIMAM Cloud Project:** OPTIMAM cloud project and buckets hosting the OMI-DB.
9. **Running of the validation**: AI Vendor project team users access was deactivated during the running of the tool. OS logs continued to be routed to the ELK stack for continual viewing by the AI vendor team during the validation. Images were retrieved by the DICOM Q/R VM from the OMI-DB and sent to the AI tool.
10. **AI Output Bucket:** AI tool processes incoming images and routes the DICOM formatted output to the DICOM Q/R VM (not always applicable) and routes the CSV output to the AI output bucket.
